# Resveratrol Promotes Proliferation, Antioxidant Properties, and Progesterone Production in Yak (*Bos grunniens*) Granulosa Cells

**DOI:** 10.3390/ani14020240

**Published:** 2024-01-12

**Authors:** Xudong Jiang, Yao Ma, Sanni Gong, Xiangdong Zi, Dawei Zhang

**Affiliations:** 1 The Key Laboratory for Animal Science of National Ethnic Affairs Commission, Southwest Minzu University, Chengdu 610041, China; 200905012004@stu.swun.edu.cn (X.J.); mayao@stu.swun.edu.cn (Y.M.); 210905012006@stu.swun.edu.cn (S.G.); 2 College of Food Science and Technology, Southwest Minzu University, Chengdu 610041, China

**Keywords:** yak, resveratrol, granulosa cell, cell proliferation, antioxidant property, steroidogenesis

## Abstract

**Simple Summary:**

The growth and development of follicles is a prerequisite for normal fertility in female animals. Granulosa cells, as an important component of the ovary, play a key role in follicular development, and their apoptosis is an important cause of follicular atresia. Resveratrol, as a common antioxidant, has various biological properties in animals in vitro and in vivo. However, the functional role of resveratrol in many aspects of yak granulosa cell activity remains unclear. Thus, we evaluated the effects of resveratrol on yak granulosa cell activity in vitro. The results showed that resveratrol when added at an appropriate concentration to the culture media of yak granulosa cells in vitro, could promote proliferation, inhibit apoptosis, enhance antioxidant properties, and promote lipid metabolism and the progesterone secretion of yak granulosa cells. This study provides some theoretical basis for research to further improve yak reproduction.

**Abstract:**

Resveratrol (RES) is a class of natural polyphenolic compounds known for its strong anti-apoptotic and antioxidant properties. Granulosa cells (GCs) are one of the important components of ovarian follicles and play crucial roles in follicular development of follicles in the ovary. Here, we explored the effects of RES on the proliferation and functions of yak GCs. Firstly, we evaluated the effect of RES dose and time in culture on the viability of GCs, and then the optimum treatment protocol (10 μM RES, 36 h) was selected to analyze the effects of RES on the proliferation, cell cycle, apoptosis, malondialdehyde (MDA), glutathione (GSH), reactive oxygen species (ROS) accumulation, lipid droplet content, ATP production, and steroidogenesis of GCs, as well as the expression of related genes. The results show that RES treatment significantly (1) increased cell viability and proliferation and inhibited cell apoptosis by upregulating *BCL-2* and *SIRT1* genes and downregulating *BAX*, *CASP3*, *P53*, and *KU70* genes; (2) increased the proportion of GCs in the S phase and upregulated *CCND1*, *PCNA*, *CDK4*, and *CDK5* genes; (3) reduced ROS accumulation and MDA content and increased GSH content, as well as upregulating the relative expression levels of *CAT*, *SOD2*, and *GPX1* genes; (4) decreased lipid droplet content and increased ATP production; (5) promoted progesterone (P4) secretion and the expression of P4 synthesis-related genes (*StAR*, *HSD3B1,* and *CYP11A1)*; and (6) inhibited E2 secretion and *CYP19A1* expression. These findings suggest that RES at 10 μM increases the proliferation and antioxidant properties, inhibits apoptosis, and promotes ATP production, lipid droplet consumption, and P4 secretion of yak GCs.

## 1. Introduction

The follicle is the site at which the female gametes develop and mature, and its development has a direct impact on the female’s fertility. This process involves the coordinated growth and development of oocytes, granulosa cells (GCs), and theca cells [1,2]. GCs secrete hormones like estradiol (E2) and progesterone (P4), as well as cytokines, which are vital for maintaining physiological rhythms and supporting pregnancy [3]. Only a few dominant follicles mature and ovulate during follicular growth and development, while most follicles degenerate via follicular atresia [4,5]. GCs are essential in determining whether follicles continue growth or undergo atresia [6].

During aerobic activities, cells generate reactive oxygen species (ROS). Excessive accumulation of ROS results in cellular oxidative stress, which is a precursor to the development of inflammation and aging (senescence) and can ultimately cause cell apoptosis [7,8]. Oxidative stress caused by an imbalance between ROS production and cellular antioxidant defense capacity is thought to be one of the factors that can trigger apoptosis in GCs and, thus, follicular atresia [9]. Malondialdehyde (MDA), the principal secondary product of lipid peroxidation (LPO) in cells, is a highly toxic molecule, and oxidative stress leads to LPO reactions that result in increased MDA in vivo [10]. Reduced glutathione (GSH) is involved in cellular physiological and metabolic actions, most notably scavenging cellular free radicals and reducing hydrogen peroxide against ROS, maintaining cellular redox levels, and protecting cells from oxidative damage [11]. It has been found that genistein can increase the antioxidant properties of GCs by attenuating ROS and MDA-enhanced GSH in GCs [12]. Lipid droplets (LDs), critical cellular organelles, play a pivotal role in lipid metabolism. They function by storing neutral lipids when there is an excess of energy and serve as energy reserves during periods of scarcity [13]. Increasing the LD content of bovine GCs affects the secretion of steroid hormones from GCs [14]. ATP serves as an energy source for cell growth and development, and more than 90% of ATP in cells originates from the oxidative phosphorylation processes in mitochondria and only a small portion from glycolytic production in the cytoplasm. Therefore, ATP is an important indicator of mitochondrial function, especially the oxidative phosphate capacity of mitochondria [15]. ATP is also a source of energy for the growth and development of GCs [16].

Resveratrol (3,4,5-trihydroxytrans-stilbene, RES) is a natural polyphenolic compound that is widely present in a variety of plants and foods, such as peanuts, mulberries, and especially in grapes and red wine [17]. RES is considered to be a natural phytoestrogen with various biological properties in animals in vitro and in vivo, including antioxidant, anti-inflammatory, anti-apoptotic, anticancer, and obesity suppression properties [18]. Recent studies have shown that RES also plays an important role in regulating animal reproduction [19,20]. Numerous signaling pathways are recognized as crucial during the development of follicles, guiding toward either luteinization and the oocyte’s release or toward follicular atresia and subsequent apoptosis [4,16]. RES has the capability to activate or inhibit these signaling pathways, thus exerting control over the physiological attributes of GCs [21,22,23]. SIRT1 plays an important role in the regulation of GC apoptosis [24]. RES, a natural agonist of SIRT1, can prevent ovarian senescence by stimulating SIRT1-related cellular mechanisms, exerting its antioxidant effects, and protecting oocytes from age-dependent defects [25]. RES also protects GCs against hydrogen peroxide-induced oxidative injury through the SIRT1 [26]. Wang et al. [27] found that in vitro maturation (IVM) medium supplemented with RES promoted bovine oocyte maturation, increased the expression levels of GSH and antioxidant genes, decreased the levels of ROS, increased cumulus cells’ (CCs) P4 secretion, and decreased E2 secretion. RES protects human GCs from induced oxidative stress [28]. It has also been reported that RES can upregulate StAR expression and promote P4 production in rat and human GCs [29,30].

Domestic yaks (*Bos grunniens*) are essential for Tibetans residing in the high-altitude regions of the Qinghai-Tibetan Plateau and surrounding areas, supplying meat, milk, and various other essentials in a harsh environment where survival of most other domesticated animal species is challenging [31]. However, yak reproductive performance is low [32]. Recent studies have shown that RES plays an important role in regulating animal reproduction [20]. The effects of RES on GCs have been studied in many mammalian species [33,34,35], but the role of RES in yak GCs is unclear. Hence, the aims of this study were to investigate the impact of resveratrol (RES) on several aspects of yak granulosa cells (GCs), including their viability, proliferation, apoptosis, cell cycle, antioxidant characteristics, lipid droplet concentration, ATP generation, steroid hormone secretion, and associated gene expression.

## 2. Materials and Methods

### 2.1. Chemicals

Triton X-100 and bovine serum albumin (BSA) were purchased from Sigma-Aldrich (St. Louis, MO, USA); fetal bovine serum (FBS), PBS, DPBS, 0.25% trypsin, DMEM/F12 medium, and pen-strep solution were purchased from Gibco (Grand Island, NE, USA). Insulin-like Growth Factor 1 (IGF1) was purchased from Sino Biological (Beijing, China); testosterone was purchased from Aladdin (Beijing, China); follicle-stimulating hormone (FSH) was purchased from Vetoquinol (Paris, France); BeyoClick™ EdU cell proliferation kit with alexa fluor 488 (C0071S), enhanced BCA protein assay kit (P10010S), cell cycle detection kit (KGA512), and cell cycle and apoptosis analysis kit (C1025) were purchased from Beyotime (Shanghai, China); Annexin V-FITC/PI apoptosis kit was purchased from Multi Sciences (Hangzhou, China); Cell Counting Kit-8 (CCK-8) was purchased from Meilunbio (Dalian, China); Oil Red O kit (G1262), GSH content assay kit (BC1175), DAPI (C0065), resveratrol (RES, R8350), MDA content assay kit (BC0025), and DCFH-DA (CA1410) were purchased from Solarbio (Beijing, China); rabbit anti-bovine FSHR antibody (AF5242) and goat anti-rabbit IgG (H+L) HRP (S0001) were purchased from Affinity (Changzhou, China); Bovine P4 ELISA Kit and E2 ELISA Kit were purchased from Jianglai Biology (Shanghai, China); ATP assay kit was purchased from Jiancheng Bioengineering Institute (Nanjing, China); 6-well plates (140675), 12-well plates (150628), 96-well plates (167008), TRIzol (15596026), PowerUpTM SYBRTM Green Master Mix (A25742), and RNA reverse transcription kit (M631) were purchased from Thermo Fisher Scientific (Invitrogen, State of California, USA).

### 2.2. Isolation and Identification of GCs

All animal procedures were conducted according to the guiding principles of the Animals Care and Ethics Committee of Southwest Minzu University (approval code: SMU-CAVS-220601001). The ovary collection, GC isolation, and culturing were performed as previously described [36]. Briefly, yak ovaries (*n* = 18, age ranging from 5 to 6 year) were obtained from a local slaughterhouse (Chengdu, China). Follicular fluid was extracted from 3–8 mm luminal follicles using a syringe, and GCs were harvested from the fluid via centrifugation (800× *g*, 5 min). All cultures were incubated at 37 °C with 5% CO_2_ using GCs up to 10^6^ cells/mL using complete medium (DMEM-F12 with 10% FBS and 1% pen-strep) unless otherwise indicated. The GCs from each yak were passaged after two or three days of complete medium culturing; we then waited for the second generation of cells to attach for 24 h and then used them for the test (within one week). The GCs were identified with immunofluorescence staining using rabbit anti-bovine FSHR antibody and goat anti-rabbit IgG. The fluorescent signals were examined under a laser confocal microscope (ZISS, LSM880, Oberkochen, Germany).

### 2.3. Effects of RES on Viability of Yak GCs

The concentrations of RES utilized were derived from prior studies on its supplementation in pig and sheep GCs [34,35]. The cell viability was determined using a CCK-8 assay in 96-well plates (5 × 10^3^ cells/well). After 24 h of cell adhesion, the cells were treated with RES (0, 1, 10, 25, 50, or 75 µM) complete medium and incubated for another 24, 36, or 48 h. Then, 10 µL of CCK-8 was added to each well in a 96-well plate, and the cells were incubated at 37 °C for 4 h under light [37]. The absorbance of each well was detected at 450 nm using an enzyme marker. The intra-group CVs and inter-group CVs of CCK-8 were 3.72% and 3.94%, respectively. The optimal concentration and time in culture were selected for the RES treatment group (RES).

### 2.4. Effect of RES on Proliferation and Apoptosis of GCs

The proliferation of yak GCs was assessed as previously described by Cheng et al. [38]. Briefly, the GCs were seeded in 12-well plates (5 × 10^5^ cells/mL). After incubation with 10 or 0 μM RES for 36 h (this optimum treatment is outlined in Section 2.3), 1 mL EdU (20 μM) solution was added to each well of a 12-well plate and incubated for 3 h. Afterward, the cells were fixed using a 4% paraformaldehyde solution for 20 min at 25 °C. This was followed by permeabilization with 0.5% Triton X-100 for 15 min and then incubation with the click-reaction solution for 45 min in a dark environment. After being washed 3 times, each well was added with a total of 450 μL DAPI (1:1000 PBS) and incubated for 15 min, then washed 3 times, and images were taken under a fluorescence microscope. The cell culture was first collected, the attached cells were washed once using PBS, and the PBS was collected, and then the attached cells were digested using EDTA-free trypsin; after the digestion was terminated, the collected cell culture, PBS, and the cell suspension remaining after the termination of the digestion were centrifuged at 300× *g* for 5 min. The supernatant was discarded, and the cells were washed twice with pre-cooled PBS. The apoptosis in GCs was identified using Annexin V-FITC apoptosis detection kits through flow cytometry (Beckman CytoFLEX, CA, USA), following the method described by Gong et al. [39].

### 2.5. Effect of RES on Cell Cycle of GCs

The GCs were seeded in 6-well plates (5 × 10^5^ cells/mL). After incubation with 10 or 0 μM RES for 36 h, the culture medium was discarded, the cells were washed with PBS, and the cells were digested with 0.25% trypsin. After termination of the digestion, the cell suspension was collected and centrifuged at 300× *g* for 5 min, the supernatant was discarded, the cells were washed twice with PBS, and the cells were fixed in ice-cold 70% ethanol at 4 °C overnight. They were then washed with PBS and then stained with propidium iodide/RNase A solution for 30 min in a dark room at 37 °C. The cell cycle of yak GCs was assessed using flow cytometry (Beckman CytoFLEX, CA, USA) as previously described by Ji et al. [40].

### 2.6. ROS Staining Assay

The ROS content in GC was analyzed by the method previously described by Wang et al. [41]. In summary, following treatment, the cells were washed thrice with PBS. Then, 10 μmol/L DCFH-DA was applied to the wells. After a 30 min incubation in darkness, the cells underwent three additional PBS washes and were stained with DAPI for 15 min. The fluorescence intensity was observed with confocal microscopy (ZISS, LSM880, Germany) at an excitation of 525 nm and emission of 460 nm. Image analysis was executed using ImageJ-win64 software (https://imagej.net/ij/ (accessed on 10 October 2022)).

### 2.7. Effect of RES on MDA and GSH of GCs

GCs were cultured in 6-well plates at a concentration of 5 × 10^5^ cells/mL. The MDA content in GC was measured following the methodology previously outlined by Zhong et al. [42]. Briefly, after incubation with 10 or 0 μM RES for 36 h, cells were collected, washed twice with PBS, and lysed, and the supernatants were extracted via centrifugation at 8000× *g* for 10 min. The MDA concentration in the supernatants was measured using an MDA assay kit according to the manufacturer’s instructions. The OD values at 450 nm, 532 nm, and 600 nm were measured using an enzyme marker (Thermo Scientific, MULTISKAN Sky 51119670, Vantaa, Finland), and the MDA content was calculated. The GSH standard curve was made according to the instructions of the GSH assay kit. The sample GSH concentration was calculated by detecting the OD value at 412 nm using an enzyme marker [43].

### 2.8. Oil Red O Staining

GCs were seeded in 12-well plates at a density of 5 × 10^5^ cells/mL. The analysis of Oil Red O staining in GC was roughly performed as described by Ran et al. [44]. In brief, after a 36 h incubation with either 10 or 0 μM RES, cells were fixed using 4% paraformaldehyde for 25 min, followed by a 15 min incubation with Oil Red O stain at 37 °C. Subsequently, the cells were washed twice with PBS, and images were captured using a microscope. After shaking for 30 min at 37 °C, the supernatant was transferred to 96-well culture plates, and optical density was measured at 510 nm using an enzyme calibrator (Thermo Scientific, MULTISKAN Sky 51119670, Vantaa, Finland).

### 2.9. Determination of ATP

The GCs were collected after incubation with 10 or 0 μM RES for 36 h. The ATP assay was performed according to the ATP assay kit’s instructions. The OD value at 636 nm was measured using the enzyme standardizer (Thermo Scientific, MULTISKAN Sky 51119670, Vantaa, Finland). The within-group and between-group CVs for the ATP kit were 3.4% and 7.21%, respectively.

### 2.10. Effect of RES on Steroidogenesis of Yak GCs

The effect of RES on steroidogenesis was evaluated as previously described [36,45]. Briefly, yak GCs were incubated for 48 h in a medium containing 10% FBS at 38.5 °C and then cultured for an additional 36 h in FBS-free medium containing 30 ng/mL FSH, 30 ng/mL IGF1, and 500 ng/mL testosterone with 10 μM RES or 0 μM RES. The culture supernatants were then collected to measure E2 and P4 concentrations using ELISA kits according to the manufacturer’s instructions. The sensitivities of the E2 and P4 assays were 7.6 pg/mL and 10 pg/mL, respectively. Inter- and intra-assay CVs were <10%.

### 2.11. Quantitative Reverse Transcription Polymerase Chain Reaction (RT-qPCR)

Total RNA was extracted using TRIzol, and the RNA D260 nm/D280 nm values were measured using a spectrophotometer (Biospec-nano, kyoto, Japan). Total RNA (A260/280 values of 1.8 to 2.0) was synthesized into cDNA using the RNA Reverse Transcription Kit according to the manufacturer’s instructions. All primers (Table 1) were designed using Primer Premier 5.0 software according to the bovine gene sequences in GenBank synthesized by Chengdu Qingke Yuxi Biotechnology Co., Ltd., Chengdu, China. RT-qPCR was performed using the Bio-Rad iQ5 and Bio-Rad iQ5 Optical System software (2.1 version, Bio-Rad Laboratories, Hercules, CA, USA) and the SYBR Green Master Mix according to the manufacturer’s protocols, adhering to the MIQE Guidelines [46]. The relative mRNA level was normalized to that of *β-actin* [36]. Three replicates per group were calculated using the 2^−ΔΔct^ method for RT-qPCR [47]. In this experiment, all *β-actin* CT values were around 19, and the CVs of the CT values for all target genes were <5.1%.

### 2.12. Statistical Analysis

Data are expressed as the means ± standard errors of the means (SEM) of three yaks (*n* = 3) with three technical replicates per animal in each experiment. Statistical comparisons were performed using Student’s unpaired *t*-test or ANOVA followed by Tukey’s multiple-comparisons test. *p <* 0.05 was considered statistically significant.

## 3. Results

### 3.1. Identification of Yak GCs

**Figure 1:**
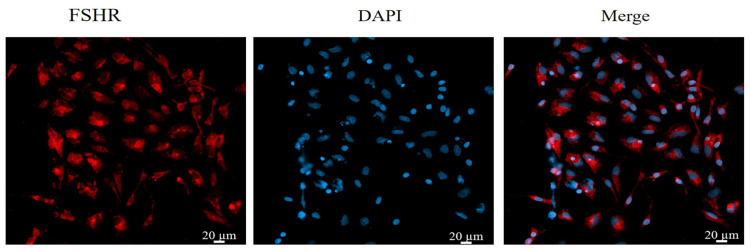
Identification of yak follicular granulosa cells via FSHR immunofluorescence. Second-generation cells were used in the experiment. Red fluorescence indicates the specific expression of FSHR, and blue fluorescence indicates the nucleus. Bar = 20 μm.

Immunofluorescence staining was used to stain and identify yak GCs because FSHR is a GC-specific expressed protein [48]. The results show that more than 90% of the cells in the field of view were positive for FSHR expression, indicating that the isolated cultured cells were GCs (Figure 1).

### 3.2. Effects of RES Concentration and Treatment Time on GC Viability

**Figure 2:**
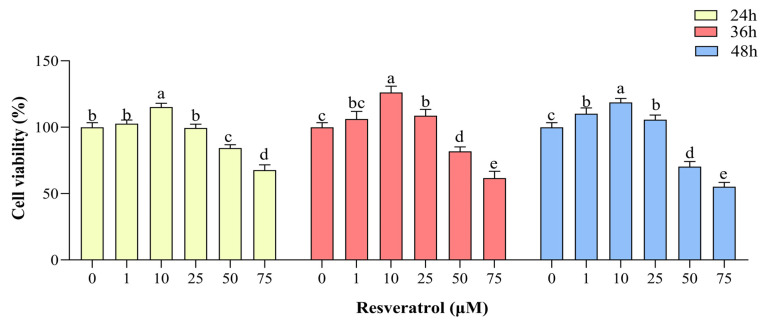
Effects of resveratrol (RES) on yak granulosa cell viability. Cell viability was determined after treatment with different concentrations of RES (0–75 µM) at 24, 36, and 48 h. Same RES treatment time between different letters indicates a significant difference, same letter indicates a non-significant difference. Results are expressed as the mean ± SEM of three yaks (*n* = 3) with three replicates per animal. Different lowercase letters indicate *p <* 0.05, two-way ANOVA (Tukey’s multiple comparison).

Compared with the control group, cell viability was increased (*p <* 0.05) in the group treated with 1 μM RES in complete medium at 48 h of treatment and in the group treated with 10 μM RES in complete medium at 24, 36, and 48 h (*p <* 0.05), with the highest cell viability seen in the group treated for 36 h. Cell viability was also increased in the group treated with 25 μM RES at 36 and 48 h compared with the control group (*p <* 0.05). GC viability was inhibited in the groups treated with 50 μM and 75 μM RES at 24, 36, and 48 h (*p <* 0.05, Figure 2). Therefore, in the subsequent experiments, the control group was treated with complete medium (89% DMEM/F12 culture + 10% FBS + 1% pen-strep), and the RES treatment group was treated with medium containing 10 µM RES for 36 h.

### 3.3. Effect of RES on Proliferation and Apoptosis of GCs

**Figure 3:**
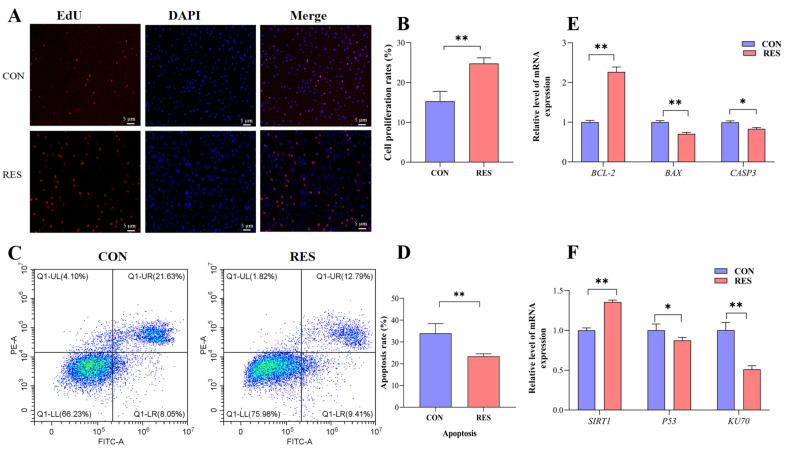
Resveratrol’s (RES) effect on the proliferation and apoptosis of yak granulosa cells (GCs). GCs were incubated for 36 h in medium without RES (CON) or with 10 μM RES (RES). (**A**) Cell proliferation was detected using EdU assay; red fluorescence indicates the EdU staining marker, and blue fluorescence indicates the nucleus. Bar = 5 μm. (**B**) Cell proliferation rate. (**C**) Apoptotic GCs detected using AnnexinV-APC/PE staining flow cytometry. (**D**) The percentages of GC apoptosis. (**E**) Proliferation and apoptosis-related genes. (**F**) SIRT1/P53/KU70 pathway-related genes. * *p <* 0.05, ** *p <* 0.01, *t*-test. The data shown are expressed as the mean ± SEM of three yaks (*n* = 3) with three replicates per animal.

The proliferation of GCs was monitored by EdU staining. Observations showed that the number of EdU-labeled red fluorescent GCs was significantly higher than that of the control group after resveratrol (RES) treatment (10 µM, 36 h) (see Figure 3A). Additionally, the cell proliferation rate showed an increase in the RES-treated group (24.78 ± 0.82% versus 15.32 ± 0.57%, *p* < 0.01, as depicted in Figure 3B). To assess the influence of RES on apoptosis in yak GCs, flow cytometry was employed. The rate of apoptosis was then analyzed using the CytExpert software (2.3.0.84, https://www.vsh.com/products/mflt/index.asp, (accessed on 8 October 2022)). The results show that RES treatment reduced the apoptosis rate compared with the control group (23.41 ± 0.67% vs. 33.96 ± 2.58%, *p <* 0.01, Figure 3D).

The expression of the apoptosis suppressor gene (*BCL-2*) was upregulated (*p <* 0.01), and the expressions of pro-apoptotic genes *BAX* (*p <* 0.01) and *CASP3* (*p <* 0.05) were downregulated with RES treatment compared with the control group (Figure 3E). The results for the detection of *SIRT1*/*P53*/*KU70* pathway gene expression show that the RES group displayed an upregulated *SIRT1* gene relative expression level (*p <* 0.01) and downregulated relative expression levels of the *P53* gene (*p* < 0.05) and *KU70* gene (*p <* 0.01), compared with the control group (Figure 3F).

### 3.4. Effect of RES on Cell Cycle of GCs

**Figure 4:**
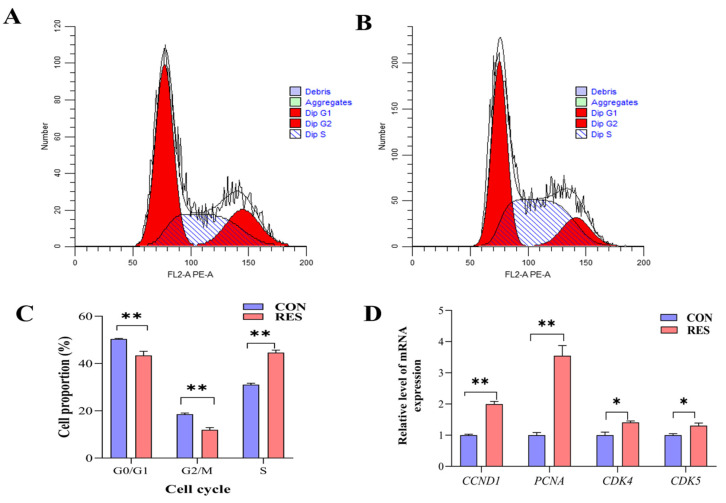
Resveratrol’s (RES) effect on cell cycle and gene expression of yak granulosa cells (GCs). GCs were incubated for 36 h in medium without RES (CON) or with 10 μM RES (RES). (**A**) The control group. (**B**) RES treatment group. (**C**) GC cell cycle percentage. (**D**) Expressions of cell cycle-related genes. * *p <* 0.05, ** *p <* 0.01, *t*-test. The data shown are expressed as the mean ± SEM of three yaks (*n* = 3) with three replicates per animal.

The cell cycle of GCs was detected using a flow analyzer and analyzed using the software ModFitLT5.1 (2.3.0.84, https://www.vsh.com/products/mflt/index.asp, (accessed on 8 October 2022)). The results show that RES treatment (10 µM, 36 h) reduced the proportion of G0/G1 (40.43 ± 1.02% vs. 50.37 ± 0.15%, *p <* 0.01) and G2/M cells (11.97 ± 0.56% vs. 18.57 ± 0.30%, *p <* 0.01), and increased the proportion of S-phase cells (44.60 ± 0.62% vs. 31.05 ± 0.36%, *p <* 0.01) compared with the control group (Figure 4C). The RT-qPCR results show that RES treatment upregulated the expression levels of cycle-related genes *CCND1*, *PCNA* (*p <* 0.01), *CDK4,* and *CDK5* (*p <* 0.05, Figure 4D).

### 3.5. Effect of RES on Antioxidant Properties of GCs

**Figure 5:**
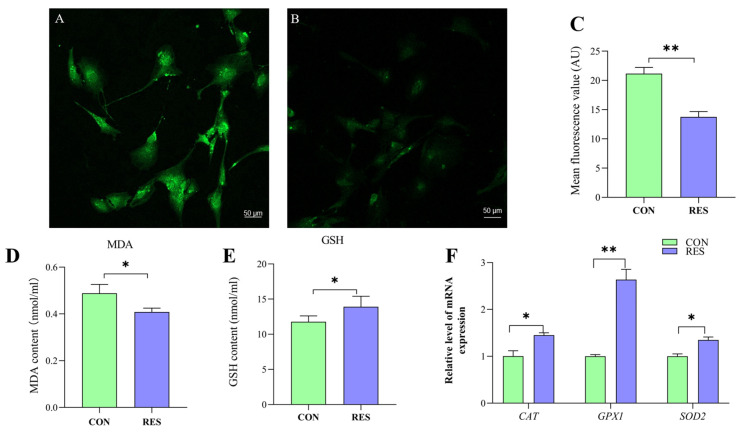
Resveratrol’s (RES) effect on antioxidant properties of yak granulosa cells (GCs). GCs were incubated for 36 h in medium without RES (CON) or with 10 μM RES (RES). (**A**) Control group. (**B**) RES treatment group. (**C**) ROS fluorescence intensity. (**D**) Concentration of MDA. (**E**) Concentration of GSH. (**F**) Effect of RES on antioxidant properties of genes. * *p <* 0.05, ** *p <* 0.01, *t*-test. The data shown are expressed as the mean ± SEM of three yaks (*n* = 3) with three replicates per animal.

To delve into the antioxidant capabilities of this compound, the ROS levels in yak GCs were assessed using the DCFH-DA fluorescent probe [35]. The control group served as a positive control (Figure 5A) for the RES treatment (Figure 5B). The ROS fluorescence intensity was analyzed, and it was found that the ROS levels in the RES treatment were significantly lower than those in the control (13.76 ± 0.92 vs. 21.15 ± 1.06, *p <* 0.01, Figure 5C).

The MDA content in the RES group was notably lower compared to the control group, suggesting that RES treatment (10 µM, 36 h) effectively reduced the MDA levels in GCs (0.41 ± 0.004 nmol/mL vs. 0.49 ± 0.009 nmol/mL, *p* < 0.05, as indicated in Figure 5D). The GSH content was detected using the GSH content assay kit, and the results show that RES treatment significantly increased the GSH level of GCs compared with the control group (13.92 ± 1.49 nmol/mL vs. 11.78 ± 0.83 nmol/mL, *p <* 0.05, Figure 5E). the RT-qPCR results show that RES treatment significantly upregulated catalase (*CAT*), superoxide dismutase 2 (*SOD2*) and glutathione peroxidase (*GPX1*) in the GCs compared with the control group (*p <* 0.05, Figure 5F). 

### 3.6. Effect of RES on Lipid Droplets (LDs) and ATP Production by GCs

**Figure 6:**
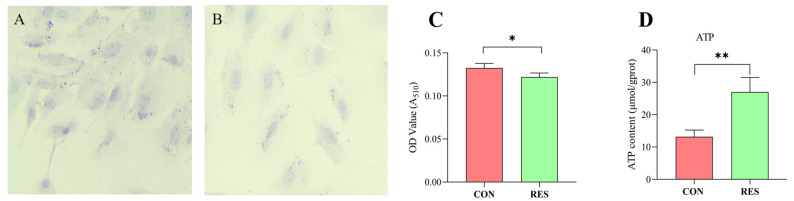
Resveratrol’s (RES) effect on lipid droplet content and ATP production in yak granulosa cells (GCs). GCs were incubated for 36 h in medium without RES (CON) or with 10 μM RES (RES). (**A**) Oil Red O staining in the control. (**B**) Oil Red O staining in the RES treatment group. Scale bars = 50 μm. (**C**) Oil Red O-stained intracellular lipids were extracted with isopropanol and quantified by measuring the absorbance at 510 nm. (**D**) RES’s effect on ATP production. * *p <* 0.05, ** *p <* 0.01, *t*-test. The data shown are expressed as the mean ± SEM of three yaks (*n* = 3) with three replicates per animal.

Lipid droplet content in GCs was determined using Oil Red O staining. The findings revealed that GCs in the control group possessed a greater number of LDs (as seen in Figure 6A) than those treated with RES (10 µM, 36 h), depicted in Figure 6B. LD quantification results indicated that the LD content in the RES group was lower compared to the control group (*p* < 0.05, shown in Figure 6C). The ATP production of GCs was measured using an ATP assay kit. The results show that RES treatment significantly increased ATP production compared with the control group (*p <* 0.01, Figure 5D).

### 3.7. Effect of RES on Steroidogenesis of GCs

**Figure 7:**
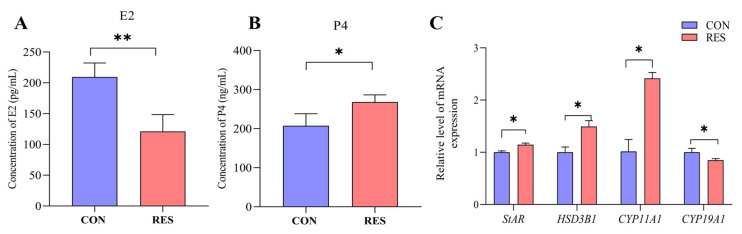
Resveratrol’s (RES) effect on estradiol (E2) and progesterone (P4) production by yak granulosa cells (GCs). GCs were incubated for 36 h in a medium without RES (CON) and a medium with 10 μM RES (RES); then, the supernatants were harvested, and estradiol (**A**) and progesterone (**B**) production were measured using ELISA. (**C**) The related gene expression. * *p* < 0.05, ** *p* < 0.01, *t*-test. The data shown are expressed as the mean ± SEM of three yaks (*n* = 3) with three replicates per animal.

The secretion of E2 and P4 by yak GCs was detected using the ELISA kit. The results show that after 36 h of incubation of the yak GCs, the E2 content in the RES treatment (10 µM, 36 h) group significantly decreased (121.08 ± 15.78 pg/mL vs. 209.50 ± 13.06 pg/mL, *p <* 0.01, Figure 7A), but the P4 content significantly increased, compared to the control group (268.12 ± 10.56 ng/mL vs. 215.29 ± 11.69 ng/mL, *p <* 0.05, Figure 7B). The RT-qPCR results show that the RES treatment significantly increased the relative expression levels of StAR, HSD3B1, and *CYP11A1* genes (*p <* 0.05) and significantly decreased the relative expression levels of *CYP19A1* (*p <* 0.05) compared to the control group (Figure 7C).

## 4. Discussion

GCs serve as an important component of ovarian follicles, and the proliferation and apoptosis of GCs are highly correlated with follicular growth and development, as well as atresia and oocyte development [49]. RES is a natural polyphenolic compound with a variety of biological properties in animals in vitro and in vivo, including antioxidant, anti-inflammatory, and anti-apoptotic effects [13]. To investigate the effect of RES on the viability of yak GCs, we detected the effects of different concentrations of RES treatment applied for 24, 36, and 48 h on the viability of yak GCs by CCK8, and we found that the effects of yak GCs were dependent on the dose of RES. Low concentrations (1, 10, and 25 μM) of RES increased the viability of yak GCs, while high concentrations (50 and 75 μM) of RES inhibited cell viability. This is similar to the results regarding the effects of RES on GCs of pigs and humans [16,34]. Studies have revealed that at low concentrations, RES functions as a natural antioxidant in normal cells, while at higher doses, it fosters pro-oxidation and triggers mitochondria-dependent cell death [50]. In this experiment, we found that the addition of 10 μM RES treatment for 36 h resulted in the greatest cell viability of yak GCs.

Apoptosis is a regulatory process of a range of related genes and proteins in cells that can occur during biological growth and development or in response to cellular stress [51]. The apoptosis of GCs affects follicular atresia and oocyte development [49]. In human GCs, it was found that the apoptosis rate was significantly reduced in the 1 μM and 10 μM RES-treated groups, and this was accompanied by the reduced expression levels of *BAX* and *CASP9* and increased the expression level of *BCL-2*; thus, RES may protect the ovarian state by affecting the apoptotic factors in human GCs [23]. Bezerra et al. [35] found that the addition of RES to sheep GC culture medium could simultaneously inhibit GCs apoptosis and promote cell proliferation by activating the PI3K pathway. In the present study, we found that after treating GCs with 10 μM RES for 36 h, there was a significant reduction in the expression of pro-apoptotic genes *BAX* and *CASP3* and a notable increase in the expression of the anti-apoptotic gene *BCL-2*. In a study by Liu et al. [52], RES was shown to ameliorate microcystin–leucine-induced apoptosis in supporting germ cells by upregulating *SIRT1*, downregulating *Bax* and *Casp3*, inhibiting *p53* and *Ku70* acetylation, and enhancing *Ku70* binding to *Bax*. Similar results were obtained in the present study, indicating that RES inhibited apoptosis and promoted the proliferation of yak GCs by affecting the *SIRT1*/*P53*/*KU70* pathway. The proliferation staining of EdU cells and apoptosis using flow cytometry further confirmed that RES significantly enhanced proliferation and decreased apoptosis in yak GCs. 

In eukaryotes, most cells go through a cell cycle to pass on their genetic material to their offspring cells. A cell cycle is divided into multiple cell cycles; the S phase of the cell cycle is where DNA replication synthesizes and assembles DNA to form chromatin and other related histones, and once the cell enters the S phase, cell division continues until the next cycle [53]. In research involving human peripheral blood endothelial progenitor cells, it was observed that the incorporation of RES markedly enhanced the cell count in the S phase, reduced the number in the G0/G1 phases, and boosted the proliferation of these progenitor cells [54]. D-type cell cycle protein (CylinD1, CCND1) is a protein that is a central component of the cell cycle regulatory machinery and binds to cyclin-dependent kinase 4/6 (CDK4/6) to regulate the G1–S cell cycle transition and trigger the expression of genes that promote S-phase entry [55]. As a prerequisite for DNA replication, PCNA is mainly found in the nucleus of proliferating cells, and its expression gradually changes with the cell cycle, i.e., a gradual increase in the G1 phase, a maximum in the S phase, and a gradual downregulation in the G2 phase and M phase, making PCNA a marker of cell proliferation and cell cycle changes [56]. Wang et al. [57] found that the addition of RES could significantly alleviate acute liver injury due to excessive APAP and promote liver regeneration by stimulating SIRT1 to reduce P53 expression, as well as by upregulating the expression of cycle-related genes (CCND1, CDK4, and PCNA) to regulate cell cycle progression. In this study, we found for the first time that the addition of 10 μM RES to the culture medium would increase the proportion of cells in the S phase, which was further verified by the upregulation of *CCND1*, *PCNA*, *CDK4,* and *CDK5* genes, indicating that a 10 μM RES treatment can promote the transition of cells from the G1 to S phase, which, in turn, promotes the proliferation of yak GCs.

ROSs, chemically reactive molecules formed during aerobic metabolism, can accumulate at high levels, resulting in oxidative stress. This stress contributes to the development of inflammation, cellular senescence, and DNA damage and can potentially lead to apoptosis [58]. Excessive levels of ROS in GCs cause a series of damages, such as destructive apoptosis, altered cell proliferation, and disrupted E2 synthesis [59]. RES reduces the ROS level and apoptotic rate of cultured GCs [26,28]. MDA represents a crucial end product of lipid peroxidation (LPO). Oxidative stress in organisms triggers lipid peroxidation reactions, leading to a rise in MDA levels and, subsequently, an increase in cellular free radicals [10]. Cai et al. [60] found in rat GCs that H2O2 increases cellular oxidative stress, and RES can mitigate cellular oxidative stress by reducing MDA and ROS levels and increasing total antioxidant capacity and SOD viability. GSH, the core of the mammalian intracellular detoxification enzyme system, has a sparse base structure that can be modified by binding to GSH. It is involved in cellular physiological and metabolic effects, most notably scavenging cellular free radicals and reducing hydrogen peroxide against ROS, maintaining cellular redox levels, and protecting cells from oxidative damage [11]. Abbasi et al. [61] found that the addition of 2 μM RES to porcine oocyte IVC for 24 h and 48 h increased GSH levels in the older group, reduced oxidative stress, and delayed oocyte senescence after ovulation. However, the effect of RES on GSH production in GCs has not been reported. 

Antioxidants, acting as free radical scavengers, play a crucial role under normal physiological conditions. Enzymes like SOD, GPX, and CAT safeguard cells from ROS and free radical harm. For instance, CAT helps reduce intracellular ROS by converting hydrogen peroxide into water and oxygen; SOD2, a key member of the superoxide dismutase family and primarily located in mitochondria, acts as the frontline defense in the antioxidant system, scavenging superoxide anion radicals and thereby shielding cells from ROS-induced damage [62]. Piras et al. [63] found that cadmium increases the level of oxidative stress in sheep IVM oocytes, and the addition of RES can increase the antioxidant level by increasing the expression of antioxidant genes *SIRT1*, *SOD1,* and *GPX1*, scavenging the excess of ROS due to cadmium exposure to restore the redox balance. The results of this study show that the addition of RES to yak serum medium could improve the antioxidant property of yak serum by decreasing the increase in MDA and the relative expression of *CAT*, *GPX1*, and *SOD2* genes, and for the first time, it was found that RES could improve the antioxidant property of yak serum by increasing the content of GSH, which could reduce the level of ROS in yak serum, thus improving the cellular oxidative stress and benefiting the survival of cells.

Lipid droplets (LDs), key cellular organelles, play a vital role in cell lipid metabolism by storing neutral lipids when energy is abundant and serving as energy reserves in times of scarcity [13]. Lipid metabolism plays an important role in animal reproduction, and studies in cattle have found that lipid metabolism in GCs is important for follicle maturation [64], while lipid metabolism by GCs is thought to be an essential source of energy for oocyte maturation [49]. In a study of bovine early embryo development in vitro, it was found that the addition of RES enhanced mitochondrial function by activating SIRT1 expression, leading to AMPK activation, and increased lipid metabolism in embryos through *β*-oxidation, thereby reducing lipid content. It was also found that RES improved bovine embryo development in vitro and improved low-temperature tolerance in bovine blastocysts [65]. The inhibition of lipid metabolism in bovine GCs decreases cell proliferation and affects follicle development [64]. Energy metabolism is pivotal in follicle development, and irregularities in ovarian energy metabolism can impact ovarian functions, encompassing oocyte maturation and ovulation [66]. Due to the low glycolytic activity of oocytes, normal follicle development depends on the uptake of glucose by GCs through glucose transporter proteins in the cell membrane, and GCs produce lactate through the glycolytic pathway to provide energy for follicle development [67]. Therefore, the amount of GCs around the oocyte is important to maintaining the ATP levels in the ovary. In human GCs, it was found that RES promoted ATP production [16,28]. To our knowledge, this study documented for the first time that RES reduces lipid droplet content and promotes lipid metabolism in GCs. ATP production was also increased, which provided more energy to promote cell proliferation.

GCs are important hormone-secreting cells in females and can secrete a number of hormones, such as E2 and P4, as well as other cytokines, which are important for maintaining physiological rhythms and pregnancy in females [3]. Steroid hormone synthesis begins with the translocation of cholesterol from the outer to the inner mitochondrial membrane via the StAR in the mitochondria through CYP11A1, the only enzyme involved in the conversion of cholesterol to pregnenolone to generate pregnenolone; this step is the slowest and, therefore, the rate of steroid hormone synthesis is controlled by this step. Pregnenolone is converted to P4 by the action of 3B-HSD [68]. CYP19A1, a key gene for E2 synthesis, transduces testosterone to E2 [69]. RES, a phytoestrogen carrying out the selective regulation of E2 receptors and aromatase inhibitors [70], would affect oocyte development and maturation by influencing hormone secretion, which, in turn, would affect oocyte maturation [71]. The dose-dependent effects of RES on stimulatory P4 secretion and inhibitory E2 secretion were observed in bovine and porcine GCs [27,72]. Another study discovered that suppressing lipid metabolism in bovine GCs decreased P4 secretion, which could potentially influence follicle growth [64]. Studies on rat and human GCs found that RES increased the levels of StAR genes and proteins and increased P4 secretion [29,30]. Another study found that 10 μM RES analog (2-hydroxy-3,5,40-trimethoxystilbene) significantly increased the secretion of P4 by porcine GCs [73]. In this study, we observed that incubating yak GCs in a culture medium with 10 μM RES for 36 h stimulated lipid metabolism and P4 secretion by upregulating the genes *StAR*, *HSD3B1*, and *CYP11A1* while simultaneously reducing E2 secretion through the downregulation of the *CYP19A1* gene. This suggests that RES facilitates the luteinization of follicles.

## 5. Conclusions

RES treatment (10 μM, 36 h) significantly increased cell proliferation, inhibited apoptosis, increased the number of S-phase cells, and improved the antioxidant capacity of GCs by decreasing MDA and ROS levels and increasing GSH levels in yak GCs. RES promoted cellular lipid metabolism by decreasing the lipid droplet content in GCs and increasing ATP production. RES increased the secretion of P4 and reduced the secretion of E2 in yak GCs. These results have improved our understanding of the effects of RES on GC proliferation and functions, providing some theoretical basis for improving yak reproduction.

## Figures and Tables

**Table 1:** Primer sequences for quantitative real-time PCR.

Gene	Sequence (5′-3′)	PCR Product Size (bp)	GenBank ID
*BCL-2*	F: TTCGCCGAGATGTCCAGT R: CCCTCCGAACTCAAAGAAGG	132	NM_001166486.1
*BAX*	F: CGAGTGGCGGCTGAAATGT R: GGCCTTGAGCACCAGTTTG	93	NM_173894.1
*P53*	F: CCCATCCTCACCATCATCAC R: GCACAAACACGCACCTCAA	80	NM_174201.2
*PCNA*	F:AGAGCTGAAGATAACGCGG R: GATCTCGGCATATACGTGCAA	181	NM_001034494.1
*CASP3*	F: GACCATAGCAAAAGGAGCAGT R: TTCTGCAATAGTCCCCTCTG	130	NM_001077840.1
*KU70*	F: AATTGACTCCTTTTGACATGAGCAT R: CCATAGAACACCACTGCCAAGA	100	NM_001192246.1
*CCND1*	F:GCCGAGGAGAACAAGCAG R: CGTCAGGCGGTGATAGGA	180	NM_001046273.2
*CDK4*	F: CACTCTGGTATCGTGCTCCA R: GGGCAGTCCAATCAGGTCAA	170	NM_001037594.2
*CYP11A1*	F: CAGGGCTCCGGAAAGTTTGT R: GGGACACTGGTGTGGAACAT	173	NM_176644.2
*SOD2*	F: ACCTCAACGTCGCCGAGG R: CCAACCGGAGCCTTGGAC	260	NM_201527.2
*GPX1*	F: CCTGAAGTACGTCCGACCAG R:GTCAGGCTCGATGTCGATGG	289	NM_174076.3
*CAT*	F: TCACTCAGGTGCGGACTTTC R: GGATGCGGGAGCCATATTCA	162	NM_001166486.1
*StAR*	F: TGGCATGGCCACACTCTATG R: GTCCTTGAGGGACTTCCAGC	111	NM_174189.3
*HSD3B1*	F: TCCGGGTGCTAGACAAAGTC R: TGACGTCAATGACAGAGGCG	171	NM_174343.3
*SIRT1*	F: CTGAAGAATCTGGTGGTGAAGTT R: TGTTAGAGGTGTCCAGCATGATG	186	NM_001192980
*β-actin*	F: CCATCGGCAATGAGCGGTTCC R: CGTGTTGGCGTAGAGGTCCTTG	132	NM_173979.3

Abbreviations: F = forward primer; R = reverse primer.

## Data Availability

Data are available from the first author, Xudong Jiang (200905012004@stu.swun.edu.cn), upon request. The data are not publicly available due to privacy or ethical restrictions.
